# Preschoolers are sensitive to free riding in a public goods game

**DOI:** 10.3389/fpsyg.2014.00729

**Published:** 2014-07-15

**Authors:** Martina Vogelsang, Keith Jensen, Sebastian Kirschner, Claudio Tennie, Michael Tomasello

**Affiliations:** ^1^Department of Developmental Psychology, Institute for Psychology, University of KasselKassel, Germany; ^2^School of Psychological Sciences, University of ManchesterManchester, UK; ^3^Department of Developmental and Comparative Psychology, Max Planck Institute for Evolutionary PsychologyLeipzig, Germany; ^4^School of Psychology, University of BirminghamBirmingham, UK

**Keywords:** prosocial behavior, moral development, cooperation, fairness, free-riding

## Abstract

Despite the benefits of cooperation, selfish individuals often produce outcomes where everyone is worse off. This “tragedy of the commons” has been demonstrated experimentally in adults with the public goods game. Contributions to a public good decline over time due to free-riders who keep their endowments. Little is known about how children behave when confronted with this social dilemma. Forty-eight preschoolers were tested using a novel non-verbal procedure and simplified choices more appropriate to their age than standard economic approaches. The rate of cooperation was initially very low and rose in the second round for the girls only. Children were affected by their previous outcome, as they free rode more after experiencing a lower outcome compared to the other group members.

## Introduction

People are remarkably cooperative, engaging in joint ventures from cooperative hunting in small groups to large-scale institutions such as elected governments. What makes this cooperation remarkable is that non-contributors can benefit from the efforts of the contributors without paying the costs. A classic example is the tragedy of the commons (Hardin, 1968) in which everyone has equal access to a shared resource. The best group outcome is if no one overutilises the resource, such as overgrazing the commons, but the best short-term outcome for each individual is to have as large a herd as possible. Every rational, self-centered individual knowing this, and suspecting that others will know this too, will focus on present gains, which are certain, rather than future gains which are not. As a result, the resource—fish populations, for example—will be depleted, with species harvested to extinction (Gordon, 1954; Clark, 1973). Public goods that cannot be depleted, so-called non-rivalrous goods, such as elected governments and public television, are still vulnerable to free-riding (Feeny et al., 1990), i.e., people can exploit investments of others while not investing themselves. In Germany, for example, there is a fee people need to pay for public television, but you can also watch it if you don't pay your fee. If a lot of people free-ride and only few pay the fee, fees will likely go up in the future. Public goods, then, pose a social dilemma between individual and group interests (Kollock, 1998); yet, despite the temptation to free-ride and the prevalence of free-riders, cooperation can, and does, exist (Ostrom, 1990).

A useful tool to probe this social dilemma is the public goods game (Marwell and Ames, 1979, 1980; see Ledyard, 1995 and Camerer, 2003 for reviews). In the public goods game, participants (players) are given an endowment (usually money). Each player can contribute a portion of this endowment to a public pot. The amount in the public pot is multiplied by the experimenter by some factor and then divided equally amongst all players, and the game is repeated over several rounds. As a result, the best group outcome is for all players to cooperate, namely to contribute their entire endowment in each round. However, the best individual strategy is to contribute nothing—to keep all of the personal endowment—while also collecting a share of the public pot. The temptation to free-ride on the contributions of others should be common knowledge, and strictly rational players should therefore not contribute anything from the first round. As a result, everyone would only get his or her personal endowment, despite the possibility of a greater benefit for everyone if all contributed. This is not what people typically do. Participants (typically Western university undergraduates) contribute about 40–60% of their endowment on the first round, but the presence of non-contributors causes a decline in contributions across rounds, while never reaching zero (the Nash equilibrium). The decline in cooperation is likely due to the fact that most people are *conditionally cooperative*, i.e., they cooperate if others cooperate as well (Fischbacher et al., 2001). In addition, after having experienced free-riding group members, conditional cooperators will also defect. Why people contribute at all in the first round is surprising to economists. How cooperation can persist in the face of free-riding is puzzling to evolutionary theorists. To psychologists, questions remain as to what motivates those who contribute and those who free-ride.

Despite the importance of social dilemmas in the evolution of human sociality (Bowles and Gintis, 2011; Tomasello et al., 2012), there has been relatively little attention devoted to how responses to these develop in early childhood. There has been, however, recent interest in social decision-making, notably fairness. For instance, infants as young as 15 months have been shown to have expectations of equitable resource distribution (Schmidt and Sommerville, 2011). Even so, 3- to 4-year-old children are predominantly self-regarding with resource distribution (Fehr et al., 2008), an effect that has been found in various cultures (Rochat et al., 2009; though see House et al., 2013b for a decrease in costly sharing through middle childhood). Sharing at 3–4 years is modulated by the ability to delay gratification (Thompson et al., 1997). Children will share with friends and strangers (Moore, 2009) at 4, but aversion to advantageous inequity (being better off than others) may not appear until about 8 years of age (Fehr et al., 2008; Blake and McAuliffe, 2011). By 9 years of age, children already show a sense of fairness like adolescents (Gummerum et al., 2008). Research has also addressed the sensitivity of children to being treated unfairly. The ultimatum game has one player (proposer) offer a distribution of resources to a second player (responder); if the responder rejects the offer, both get nothing (Güth et al., 1982). Children as young as 4 have been tested, and the basic finding is that fair offers increase with age, and may be related to the development of false belief understanding Takagishi et al., 2010) as well as a decrease in impulsivity (Steinbeis et al., 2012; see also Harbaugh et al., 2007; Sutter, 2007; Kogut, 2012). Of particular interest is the responder's decision; it is the threat of rejections that drives fair offers. Five-year-olds are sensitive to fairness, and this appears to be due to expected norms of parity, but not to relative outcomes or the intentions to lead to these as in adults (Wittig et al., 2013; based on Falk et al., 2003). It is in this period of early childhood, around 5 years of age, that children start to robustly show sensitivity to fairness and an understanding of strategic decision-making in economic game contexts. This is also the period before formal schooling (in some countries, such as Germany), where children will not yet be exposed to conventionalized rules for solving social decisions (e.g., taking turns, sharing equally), which enables them to cope simply by conforming to rules rather than by thinking things through for themselves.

The first public goods study, in fact, was not on adults, but on teenagers aged 15–17 (Marwell and Ames, 1979). Participants could decide to invest tokens, representing an initial endowment of $5 USD, in either their own fund or a public fund that would be divided according to the investment decisions of the other participants. Despite assurance of anonymity (subjects were tested over the telephone) and the use of real money, public contributions were higher than expected (around 50%) despite the opportunity for rational free-riding, but lower than the optimal amount of 100%. Simplifying the returns from public contributions (provision point), increasing the amount of money at stake, and retesting individuals to increase experience had no appreciable effect on free riding (Marwell and Ames, 1980). In a later study that included teenagers, List (2004) tested a “young cohort” (under 19 years of age) as well as adults, over repeated rounds using trading cards as a resource, and found the standard level of initial contributions (though lower in the younger cohort than the two adult cohorts), with a less pronounced decline in contribution across rounds.

The first test on children had 6- to 12-year-old children in groups of six play a public goods game over ten rounds (Harbaugh and Krause, 2000). In contrast to what has been found with adults (namely a steady decrease in public contributions from an initial level of about 50%), contributions first increased, then leveled off and later slightly decreased. Age proved to be a significant factor: older children were initially more generous, but also learned to free ride more quickly than younger children did. In a similar age group (7- to 10-year-olds), Alencar et al. (2008) investigated the influence of a number of factors on the cooperative behavior in the public goods game: gender, group size and information about the number of sessions to follow (so that children either knew they were playing eight rounds or were kept uninformed about the number of rounds). Only group size significantly affected contributions (small groups of five to seven players cooperated more than large groups of more than twelve players). Peters et al. (2004) tested children (from 9 to 16) with their parents and with strangers. Children contributed about 50% of their money, but they contributed less than their parents did, and did not show an expected bias toward contributing more when playing with their parents than with strangers (“rotten kid theorem”; Becker, 1991). Finally, in a prisoner's dilemma, which is in effect the two-player-version of the public goods game, Fan (2000) found an increase in cooperation in children between 6 and 11 years of age, and also more verbal justifications in older children on the reasons behind their choices.

To gain further insights into the development of cooperation—and free-riding—in public goods games, it is important to test children as early as possible (before formal schooling), yet late enough that they understand strategic decision-making in game contexts. To address this gap in the literature, we tested 5-year-old children in a mini-public goods game. Mini-games are reduced form, or simplified games. They reduce a range of choices to a binary choice, and have produced similar results to full-form games (e.g., mini-ultimatum game; Falk et al., 2003; Wittig et al., 2013). The chief innovation of this study was that procedure was non-verbal and therefore more appropriate for younger children. It is not entirely clear that younger children in the previous public goods studies fully understood the full range of outcomes, particularly the multiplication of public contributions. Generosity and the absence of free-riding might have been due to lack of task comprehension rather than prosocial preferences. Using the same type of verbal testing as used in studies on adults is likely too difficult for younger children; it is not clear, for example, how well they understand the use of terms such as “private” and “public.” To counter this concern, we designed a novel apparatus and procedure that suits the competencies of 5- to 6-year-old children. To further ensure that they could easily follow the contribution rules of a public goods game and react accordingly to the inherent social dilemma, we designed an experiment that only presented two options for contributing (all or nothing), contained a simple physical apparatus that created the outcome “live,” and provided a highly limited number of possible group and private outcomes, all of which the children experienced during a previous familiarization phase. The apparatus allowed the children to choose outcomes for themselves and others with minimal verbal instruction and no reference to game strategy. The advantage of using an apparatus with clear contingencies rather than standard verbal game instructions is that the cognitive demands are lower. Children could therefore focus on the outcomes resulting from their actions rather than holding in mind the hypothetical outcomes that are needed in planning. Apparatus-based studies have been successfully used on younger children in economic experiments such as the ultimatum game (Takagishi et al., 2010; Wittig et al., 2013) and non-human primates (e.g., Jensen et al., 2007). The apparatus used here is modified from a study on peer helping in 4-year-old children (Kirschner and Tomasello, 2010). Furthermore, like Alencar et al. (2008), we used food rewards instead of money since food has a clear value as a commodity (Lucas and Wagner, 2005).

With this approach, we wished to see if preschool children will act like adults in a four round mini-public goods game. Specifically, we wanted to see if children would initially behave cooperatively, namely by donating their endowment on the first round. Second, would children respond conditionally in subsequent rounds, namely by decreasing their likelihood of cooperation if others did not cooperate? Our expectation was that with a more age-appropriate procedure, preschool children, like adults, would initially cooperate then quickly learn to free-ride on the contributions of others.

## Methods

### Participants

Children whose parents had previously given consent were recruited from and tested in 10 kindergartens in a medium-sized city in Germany. Children were selected at random from this list for participation in this study. Forty-eight children of 5–6 years of age took part in this study (24 girls, 24 boys), which made up twelve same-sex groups of four children each. Each group was composed of children from the same kindergarten. The children's age ranged from 66 to 76 months with a mean age of 70 months (standard deviation 2.96 months). The children came from mixed socio-economic backgrounds.

### Study design

Twelve groups of four children each were tested on two consecutive days. On the first day, the group was familiarized individually with the general procedure and on the second day (1–2 days later), they were tested in groups. The task was established as a “distributing gumballs game”. We avoided terms such as “sharing,” “cooperate” and “public goods” to avoid priming the children, and also because we could not assume that they fully understood these terms. Every trial consisted of three phases: the distribution phase, the collecting phase, and the evaluation phase. In the distribution phase, children could use an apparatus to distribute resources (gumballs) to themselves or the group. In the collecting phase, children would place the resources in the corresponding collecting containers. Finally, in the evaluation phase, children accumulated their resources in their evaluation tubes, and information on outcomes was shared as a group. Consistent with economic experiments, there was no use of deception in this study: the children played against other children for real resources under conditions of full anonymity; there was no opportunity for them to doubt the integrity of the study, as could potentially be the case when they play against absent partners (e.g., Fehr et al., 2008).

### Study materials

Gumballs were used because their round shape allowed them to roll down the ramps in the apparatus. They were also attractive to the children. However, for hygienic reasons, as well as to satisfy parental preferences, we replaced the gumballs with gummy bears when giving the rewards to the children at the end of the game. The gumballs, then, served as in-kind tokens, and because both were sweets, they would have presented similar inhibitory control issues (as opposed to using a non-food token for food).

**Figure 1:**
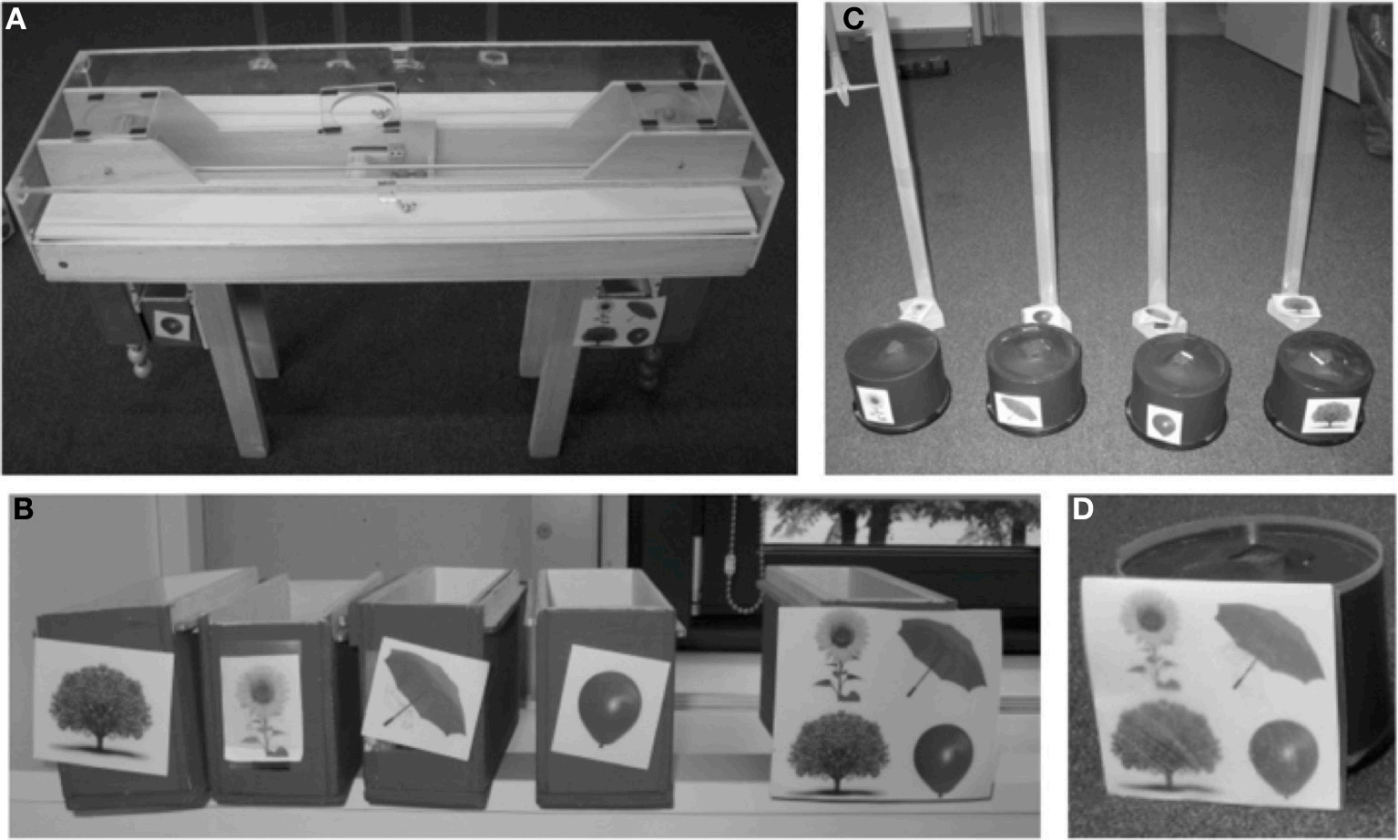
**(A)** Distributing apparatus (distributor). Two ropes can be pulled, resulting in gumballs to roll off the apparatus into private or public boxes. Choosing the private option causes two gumballs to fall into the private collection box (shown on the left) and two gumballs to fall into a trashcan (not shown). Choosing the public option causes all four gumballs to fall into the public box (right). **(B)** The four private boxes with a different symbol for each child and the public box with all four symbols. **(C)** Private collecting containers held accumulated rewards that would be put into evaluation tubes (background), allowing easy visual determination of each child's payoffs. **(D)** One of four identical public collecting containers.

The first part of the game involved a distributing apparatus (distributor) that dispensed gumballs into boxes (Figure 1A). The distributor was a wooden table (88 × 65 × 30 cm) covered with a Plexiglas lid to prevent direct access to the gumballs inside. Emerging from the sides of the length of the table were two ropes. Pulling either rope caused a slider to move any gumballs on it to the ends of the table, and at the same time caused any gumballs on a platform to be tipped off. The gumballs would then drop through holes into either a box or a trashcan. The apparatus was designed to make it obvious that choices of either the private or public side were mutually exclusive and that the non-chosen gumball became unavailable. The spring-loaded slider would return to the starting position; this not only allowed for easy rebating after each trial, but also made it impossible for anyone to see which rope had been pulled. Each child had his or her own “private” box, distinguished by a picture (tree, flower, umbrella, balloon), and only knew the identity of his or her own box; this assured subject anonymity in choices made (Figure 1B). Another box had all four images on it (“box for everybody”), and this was the public good. Gumballs that fell into the trashcans went to no one. If the rope on the “private” side was pulled, the two gumballs moved on the slider to the private side of the apparatus and fell into the private box, whereas the two gumballs on the platform on the “public” side fell into the trashcan^1^. Pulling the rope on the public side caused the two gumballs on the slider to move to the public side, and these, plus the two on the platform, fell into the public box; none went to the private box. The apparatus was designed such that either side could be public or private, and this was counterbalanced across subjects to avoid any potential side preferences. In short, the children could choose a private outcome that resulted in two rewards falling into their private box alone, or a public outcome that resulted in no rewards going into their private box, but four gumballs going to the public box (so their investment of two gumballs was doubled), resulting in one gumball for each participant.

The accumulated gumballs would be transferred from the private and public boxes into collecting containers. These were eight plastic containers with small slits at the top and clear plastic tubes inside (Figure 1C). Four of them were public containers and were labeled with all four symbols corresponding to the public container (Figure 1D) and four of them were individually labeled with a tree, flower, umbrella or balloon, according to the individual boxes (Figure 1C). The eight containers could be opened, leaving the gumballs visibly stacked in transparent plastic tubes, allowing for quick and easy comparison of the amounts in each. Additional study material consisted of a curtain that was used as a visual barrier between the distributing apparatus on the one side and the private collecting boxes and the evaluation tubes on the other side to assure anonymity of choices.

### Procedure

#### Familiarization

Familiarization took place on the first day. Children were brought from their nursery in groups of four. They were first introduced to the apparatuses and procedure as a group by E1. Half of the groups were shown public choices first and half were shown private choices first. The children saw all possible outcomes that could arise when all children cooperate (choose the public box) to where none do, with all intermediate options (Table 1).

**Table 1:** **Overview of the possible payoffs for a single round**.

**Player 1 chooses public**			**Player 1 chooses private**		
**Number choosing public**	**Number choosing private**	**Player 1 payoff**	**Number choosing public**	**Number choosing private**	**Player 1 payoff**
3	0	4	3	0	5
2	1	3	2	1	4
1	2	2	1	2	3
0	3	1	0	3	2

Four children could choose between the public option and the private one. Payoffs (number of gumballs) for each player depended on the choices made by the other three players. Here, all possible outcomes for one round for one player are shown on the basis of whether he or she chose the public outcome, and the number of players choosing public or private. The highest possible payoff in a round was five gumballs and the lowest was one.

In the individual familiarization, one child stayed with E1 while the others left the room with E2 where they drew pictures (this kept the children from discussing the game amongst each other). E1 assigned one private box to the child, stressing that he or she was not supposed reveal to the others which box was his or hers. Hence, only the child and E1 would know which box was his or hers. The private and public boxes were attached to the distributor. The child followed E1s instructions as to which rope to pull after the distributor was baited, and then answered how many gumballs fell into the respective boxes as a result of his or her action. That is, children were asked to pull the rope to the private and public sides over the course of the familiarization phase and to comment of the results of these actions. After each choice, the child detached both boxes from the distributor and then put the gumballs into the appropriate collecting containers. E1 explained that the child would get all the gumballs that he or she accumulated in the private container and that the gumballs would be distributed evenly among the four public containers, one of which would go to the child. After each child had his or her turn, the evaluation phase started. All four children returned to the room with E2, who was blind to which symbol belonged to which child and to which direction they had pulled. E1 left the room. Now, children saw the effect their decision had on their own and the others' outcomes. First, the public containers were opened. E2 explained to the children what these outcomes meant (for example, how many children had chosen to pull the private side). Then, the private containers were opened. The content of the public and private containers were put into the evaluation tubes, allowing everyone to see how many gumballs they and the others got, without knowing whose tube was whose apart from their own. Outcomes were never described in normative or moral terms such as right or wrong, good or bad. At the end of each familiarization trial, E2 asked one of the children how the outcomes had come about, specifically, whether the number of gumballs in each collection tube was the result of either a public or private choice (“Where did the gumballs come from?”). Children were generally able to do so. Only a few required additional prompting, namely by comparing the amounts in the different tubes (i.e., the tube that had less was from a child who chose the public option and the one that had more was from a child who chose the private one).

At the end of the familiarization, the group was told that today had just been practice and that on the next day, they would get to come again and make their own choices and get to take the rewards home with them.

#### Testing

The four test rounds were carried out one or two days after the familiarization. The study setup was kept the same, except that the private collecting containers and the evaluation tubes now stood behind the curtain; as well, the evaluation tubes were occluded by an opaque bar to ensure that choices were anonymous. All children carried out their decision in the testing room together with E1 while the other children waited outside with E2.

Before the test started, each child was asked whether he or she still remembered how the apparatus and procedure worked and was then asked to demonstrate the correct use of the distributor, collecting containers and evaluation tubes. Sixteen children (33%) were initially unable to explain the apparatus. Specifically, while they recalled the features of the apparatus, i.e., that there was a private and public side, they sometimes forgot how many gumballs were involved and hence needed an additional demonstration of at least one side of the distributor by the experimenter. Children were then able to recall what happened to the gumballs. E1 pointed out the occlusion of the collecting containers and evaluation tubes and the anonymity that this ensured. E1 also reminded the child that this was not a practice, and that they would all take their rewards home with them at the end.

After this reminder, E1 initiated the first distribution round by telling the child that today he or she would get to decide where he or she wanted to pull and that no one but the child would know what he or she chose. E1 then stood aside in another corner of the room with her back turned. After choosing, the child announced he or she was done and E1 returned and guided the child to put the gumballs from the target boxes into the designated collecting containers. Finally, the experimenter returned the boxes back to the table or shelf.

After each child had had his or her turn, all four children returned into the room for the evaluation round. However, unlike the familiarization day, the private collecting containers were kept closed and only the public collecting containers were opened by E2. One by one, the children took one of the public collecting containers, went behind the curtain, and put the gumballs from their private collecting container plus their share from the public container into their evaluation tubes. Because this evaluation phase took place in private behind the curtain, the other children could not know how much each child had received. However, each child could conclude from the public collecting containers how many children had contributed to the public good. Children sometimes needed assistance with opening their private collecting containers and placing the gumballs into the evaluation tubes, so E1 sat behind the curtain and could help them. After each child had put his or her gumballs away, all four children left the room with E2 and a new round started. In total, four rounds were completed.

## Coding and reliability analysis

All of the children's actions were videotaped and their decision (pulling to their private target box or to the public target box) was coded from video by E1 with 0 corresponding to a choice of the public side and 1 to a choice of the private side. A randomly selected sample of 25% of trials (3 groups) was analyzed by a second evaluator for choices (private vs. public). Interobserver reliability was perfect (Cohen's κ = 1).

### Statistical tests

To test whether children's choices of the private or public option would depend on the outcome they had observed in the previous round, we used a Generalized Linear Mixed Model (GLMM; Baayen, 2008) with fixed effects of gender, familiarization order, round and previous outcome, and random effects of individual and group membership.

Further, we used non-parametric tests, i.e., Cochran's Q and McNemar's change tests. Cochran's Q tested for significant changes in the choice behavior across the four rounds of the game, whereas the McNemar test compared the behavior between two consecutive rounds. As the latter is a change test, children who chose the same option in two rounds were excluded from this *post-hoc* analysis. To test whether gender and type of familiarization had an effect on the children's choice behavior, we used a Mann-Whitney-Test. All statistical analyses were 2-tailed and assumed an alpha-level of *p* < 0.05 for significant results.

## Results

All children passed the familiarization phase. The results of the GLMM showed that gender and round had an effect on the number of choices for the private side, with boys choosing the private side more often (estimate ± *SE* = 1.45 ± 0.66, *z* = 2.21, *p* = 0.027) and the amount of private choices increasing over the course of the test (0.04 ± 0.02, *z* = 2.08, *p* = 0.038). Most importantly, the decision for either the private or public side was determined by the previous outcome: having a worse outcome then the rest of the group members led to a decision for the private side (−1.45 ± 0.40, *z* = −3.65, *p* = 0.0003). There were no interaction effects (see Table 2). Random effects were controlled (individual, variance = 2.34; group membership < 0.001).

**Table 2:** **Overview of the GLMM analysis**.

**Term**	**Estimate**	***SE***	***z***	***p***
(Intercept)	−0.188	0.714	−0.263	0.793
Previous outcome	−1.451	0.397	−3.650	< 0.001
Gender	1.454	0.658	2.209	0.027
Familiarization	0.352	0.663	0.531	0.595
Round	0.036	0.018	2.080	0.038
Gender ^*^ Round	−0.030	0.040	−0.700	0.486
Previous outcome ^*^ Gender	0.590	0.800	0.730	0.464

There were only main effects for previous outcome, gender and round. Hence, children's choices of the private side were influenced by their outcome in the previous round (coded as difference between the amount they had obtained and that of the other group members, being worse off than other group members increased choices of the private side), their gender (boys tended to choose the private side more often), and which round was played (choices of the private side generally increased). Familiarization, i.e., whether the private or public option was demonstrated first did not influence the children's choices. Also, there were no interaction effects of gender and round or previous outcome and gender.

**Figure 2:**
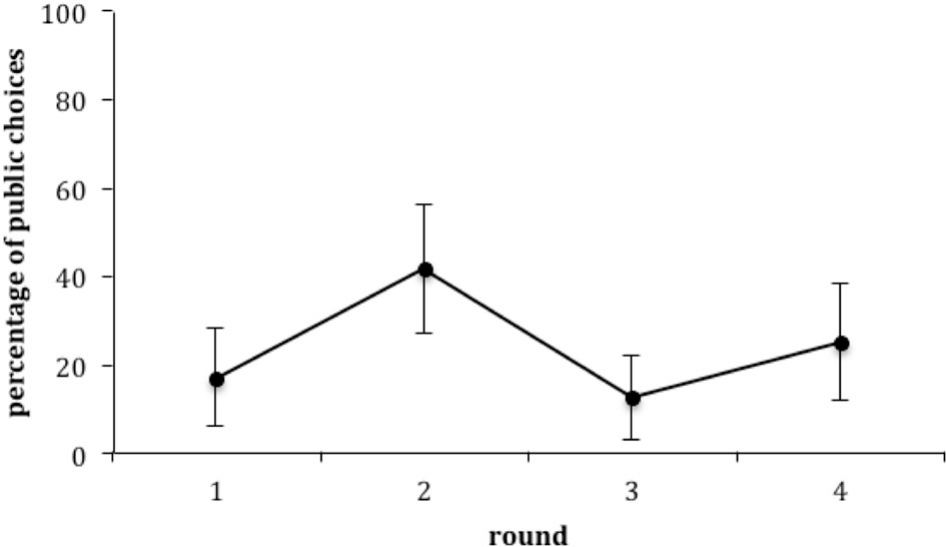
**Percentage of choices of public outcome for the four rounds of the game (mean ± 95% CI)**.

*Post-hoc* analyses show that there was no effect of order of presentation of public or private choices during familiarization (Mann-Whitney-Tests, Round 1: *z* = −1.533, *p* = 0.245, Round 2: *z* = −1.159, *p* = 0.38, Round 3: *z* = −0.864, *p* = 0.666, Round 4: *z* = −0.66, *p* = 0.74). Choices of public vs. private outcomes differed across the four rounds of the experiment (Cochran's *Q* = 13.269, *p* = 0.004, *N* = 48, *df* = 3). From the McNemar's change test, children chose the public side more often in the second round than the first (*N*_Round 1 only_= 20, *N*_Round 2 only_= 8, *p* = 0.036). In the third round, they chose the public side less often than in the second (*N*_Round 2 only_= 5, *N*_Round 3 only_= 19, *p* = 0.007), while the last round (round 4) did not differ from the third (*N*_Round 3 only_= 10, *N*_Round 4 only_= 4, *p* = 0.18). First round choices were 17% public, peaked at 42% in round 2 and ended at 25% (Figure 2).

**Figure 3:**
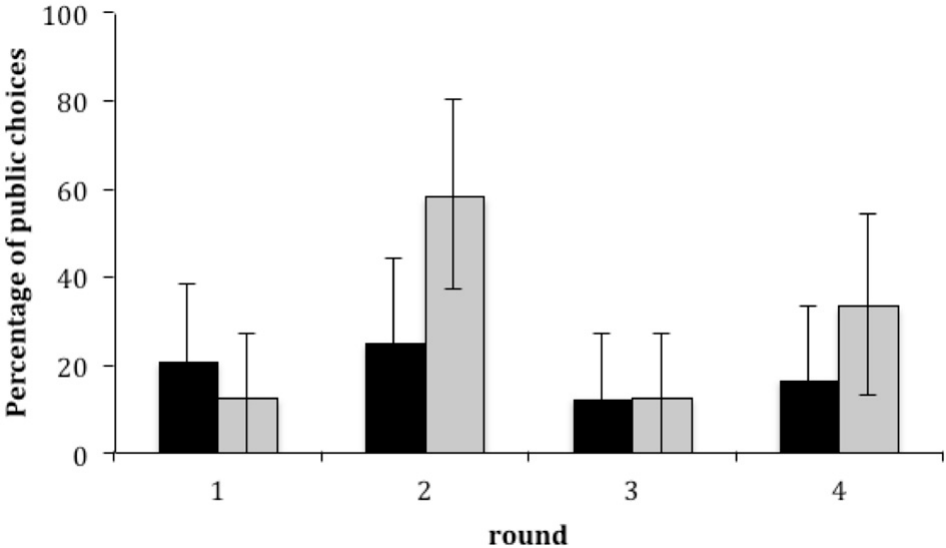
**Percentage of choices of public outcome for the four rounds of the game for boys (black bars) and girls (gray bars) shown as mean ± 95% CI**.

A *post-hoc* analysis of gender showed that boys chose the private option more often than the public one, and did so equally often in each round (range = 75–88%; Cochran's *Q* = 1.5, *N* = 24, *df* = 3, *p* = 0.795). Girls, unlike boys, did sometimes make public donations (range = 12–58%), primarily by choosing the private option less often in the second round (Figure 3). Chi-square analyses showed that boys always preferred the private option over the public option: Round 1: χ^2^ = 8.167, *df* =1, *p* = 0.007; Round 2: χ^2^ = 6, *df* = 1, *p* = 0.023; Round 3: χ^2^ = 13.5, *df* = 1, *p* < 0.001; Round 4: χ^2^ = 10.667, *df* = 1, *p* = 0.002. Girls, on the other hand, only preferred the private option in Rounds 1 and 3: Round 1: χ^2^ = 13.5, *df* = 1, *p* < 0.001, Round 2: χ^2^ = 0.667, *df* =1, *p* = 0.541; Round 3: χ^2^ = 13.5, *df* = 1, *p* < 0.001; Round 4: χ^2^ = 2.667, *df* = 1, *p* = 0.152. Overall, boys and girls show different choices: Cochran's *Q* = 15.375, *N* = 24, *df* = 3, *p* = 0.001; McNemar tests confirm that the amount of private choices in Round 2 differs from Round 1 and 3, while girls chose the private option equally often in Rounds 3 and 4; *N*_Round 2 only_ = 3, *N*_Round 1 only_= 14, *p* = 0.013; *N*_Round 2 only_ = 3, *N*_Round 3 only_= 14, *p* = 0.013; *N*_Round 3 only_= 7, *N*_Round 4 only_= 2, *p* = 0.18.

## Discussion

When presented with a simplified version of the public goods game, children made strategic choices. Five year-old children—the youngest yet tested in a public goods game—were, to some degree, conditional cooperators. These results are consistent with House (2013a) who found contingent cooperation in children beginning at 5.5 years of age. Adults playing the public goods game typically contribute 40–60% of their endowment in the first round of play, then reduce their contributions to near zero by the end of the game in response to selfish free-riders (Camerer, 2003). Children in our study initially started out with low contributions, but then increased these in the second round to what is typically seen in adults in the first round, before reducing their contributions to the public good. Some children, then, free-ride while others adjust their contributions conditionally. We did not find the steady decline in contributions over the four rounds; more trials—in adults, there are typically 10 rounds—would be needed to see if children became consistently more selfish over time. Children only had the opportunity to play four rounds due to time constraints; it was time-consuming to make choices for four players successively. Future studies could have multiple apparatuses, allowing children to make simultaneous choices. It may be the case that some children were signaling a willingness to cooperate in a manner consistent with generous tit-for-tat which is an evolutionarily stable strategy in repeated play in a prisoner's dilemma (Nowak and Sigmund, 1992). It may also be that children, who would not have encountered strategic interactions of this sort before, were still exploring the structure of the game to determine what strategies the others were using.

Gender differences typically do not emerge in economic studies on adults, but there are exceptions, such as men being more likely to punish out of principle (Eckel and Grossman, 1996), women under 50 donating more to charity than their male peers (List, 2004) and men being more likely to signal their tendency to defect in a prisoner's dilemma while women signal cooperativeness when being observed (Charness and Rustichini, 2011). While relatively small sample sizes make it difficult for us to draw firm conclusions, we found that boys and girls played the mini-public goods game differently. Boys were consistently selfish in their contributions from the first round to the last, whereas girls behaved in a manner more consistent with contingent cooperation, specifically generous tit-for-tat. Gender differences have shown up in other studies. For instance, boys more than girls were more sensitive to group membership when responding to disadvantageous inequity (Fehr et al., 2008), although in another study, while girls were more prosocial, sexes did not differ in their contingent reciprocity (House et al., 2013a). Boys and girls tend to interact in same-sex groups, leading to different subcultures with different types of play and ways of communicating (Maccoby, 2002). Interactions between girls generally focus on interpersonal closeness, nurturing, and talking, with boys' play being more task-oriented and competitive. The gender differences in this study reflect differences in preschoolers' play behavior. Boys seemed to have interpreted the task as a competition game, while girls seem to have interpreted it as a cooperative one. (Girls tend to be more prosocial in resource distribution studies; e.g., Gummerum et al., 2008; Blake and Rand, 2010; House et al., 2013a). Both boys and girls played the mini-public goods game strategically, albeit with different strategies.

Reducing the game to binary choices (public vs. private), using an apparatus that made the outcomes of choices visible, while assuring subject and experimenter anonymity allowed us to find both a willingness to cooperate as well as a conditional response when confronted with free-riders. The fact that these results reflect what is seen in studies on adults suggests that these tendencies appear earlier in development than had previously been found using verbal instructions akin to standard economic experiments on adults. Harbaugh and Krause (2000) found that only older children learned to free-ride, and that there was a general rise in contributions over 10 rounds, contrary to studies on adults. As they suggest, it may be that the generous contributions may have been due to mistakes rather than altruistic tendencies. Alencar et al. (2008) did find free-riding and a concomitant decline in cooperation in children with a mean age of 8 years in large groups (more than 12 children) but not in small groups (5–7 children). By simplifying the task demands in our study, notably by using an apparatus-based approach that has been successful in other studies of fairness and prosociality in children (e.g., Kirschner and Tomasello, 2010; Wittig et al., 2013) as well as great apes (e.g., Jensen et al., 2007; Kaiser et al., 2012), we were able to get the children to understand—and demonstrate an understanding—of the consequences of their actions for themselves and for the group. Children might still have made mistakes; they might have also been “testing the waters” to see what others would do. But the fact that participants—notably boys—did free-ride and that girls, at least, did respond conditionally to this, despite a willingness to cooperate, suggests that by 5 years of age, children—in a Western, industrialized society, at least (Henrich et al., 2010)—are capable of conditional cooperation as well as free-riding (see also House et al., 2013a).

Future research could use a similar non-verbal approach to test great apes and other species to determine whether these competitive and cooperative tendencies appear earlier phylogenetically. This approach could also be applied to children in other parts of the world where terms such as “public” and “private” may not be understood in the way they are in a Western country; to date, little work on social decision-making has been done cross-culturally (Rochat et al., 2009; Zebian and Rochat, 2012; House et al., 2013b). It might also be possible to test even younger children to better ascertain when social preferences and strategic decision making emerge. One important innovation that could be applied in future studies would be to add a punishment option since in adults, at least, this effectively discourages free-riding (Fehr and Gächter, 2002). We maintained anonymity in this study, but it would be valuable to allow children to know what the others contribute to see if reputation positively influences cooperation (Milinski et al., 2002). It is not immediately obvious that these factors would influence children. For instance, in a mini-ultimatum game in which children could choose between selfish outcomes or alternatives of varying degrees of fairness, 5-year-olds were more selfish and less strategic than adults, despite sitting next to each other (Wittig et al., 2013). Streamlining the paradigm will be important for future work, so that more trials can be conducted over a shorter period of time. This could serve to heighten the competitive elements of the game while reducing demands on the children's patience, and it would allow more rounds to be conducted to better determine whether children reach equilibrium. Children could also be tested in same sex groups and have these results contrasted with mixed-sex groups to better determine what role, if any, gender plays in social dilemmas.

Already by 5 years of age children will have learned to share (e.g., Moore, 2009), are averse to disadvantageous inequity (e.g., Wittig et al., 2013), but are not yet averse to advantageous inequity (Blake and McAuliffe, 2011). Children will have learned norms of sharing when in pairs, but will likely have had less experience and less instruction on how to interact in groups, particularly when decisions are private though outcomes are not. It is not surprising that children explore their options, but it is impressive that they learned the game as quickly as they did, particularly by free-riding early on. The children did appear to understand the strategic nature of their choices, namely that the amount they received depended on what the others did. The ability to engage in strategic social interactions—to the detriment of the group—is already evident by 5 years of age. The ability to respond contingently to non-cooperators, and to free-ride on others, allows humans to cooperate in large groups, and yet fail spectacularly to do so even when it is in the best interests of the group (as in the tragedy-of-the-commons; Hardin, 1968). Whether this ability—and shortcoming—is uniquely human remains to be seen. To answer this question, the nonverbal approach to the public goods game, as used here, might be suitable for testing on our closest living relatives.

### Conflict of interest statement

The authors declare that the research was conducted in the absence of any commercial or financial relationships that could be construed as a potential conflict of interest.

## Footnotes

^1^ The terms “private” and “public” were never used to avoid confusing or priming the children. Instead, they were referred to as belonging either to the child or to everybody.
